# Toward almost closed genomes with GapFiller

**DOI:** 10.1186/gb-2012-13-6-r56

**Published:** 2012-06-25

**Authors:** Marten Boetzer, Walter Pirovano

**Affiliations:** 1 BaseClear BV, Einsteinweg 5, 2333 CC, Leiden, The Netherlands

## Abstract

*De novo *assembly is a commonly used application of next-generation sequencing experiments. The ultimate goal is to puzzle millions of reads into one complete genome, although draft assemblies usually result in a number of gapped scaffold sequences. In this paper we propose an automated strategy, called GapFiller, to reliably close gaps within scaffolds using paired reads. The method shows good results on both bacterial and eukaryotic datasets, allowing only few errors. As a consequence, the amount of additional wetlab work needed to close a genome is drastically reduced. The software is available at http://www.baseclear.com/bioinformatics-tools/.

## Background

*De novo *sequencing of unknown species and variants has become a major application of next-generation sequencing (NGS) technologies. As a consequence, there is a high demand for *de novo *assembly tools that can reconstruct the genomic sequence from millions of short reads. At present a vast number of tools is available to create a draft assembly that represents the genome in a number of contiguous sequences (contigs), such as Velvet [1], ABySS [2], and SOAPdenovo [3]. Nonetheless, the full closure of a complete genome remains a challenging and often expensive task. Generally, problems reside in the assembly of low-coverage areas and repetitive elements. Even though the distance information of paired-read sequences can help to bridge these difficult areas by linking the contigs into larger scaffolds [4,5], scaffolding does not solve the inherent problem of low-coverage and repetitive elements: it does not add novel sequence information to the draft assembly. At present, two strategies have been proposed to automatically close draft assemblies: Li *et al*. [3] included a gap closure routine to their SOAPdenovo assembly software, whereas more recently Tsai *et al*. [6] introduced the IMAGE algorithm. Both methods use De Bruijn graphs to create a local assembly and seek to fill the gaps with the resulting contigs. A drawback of these strategies is that no prior knowledge of the estimated gap size is taken into account. Therefore, the local assembly might not reflect the true genomic situation. Also, the practical usability of these methods by a broad audience requires additional development. As a consequence, at present, most genome assemblies are still incomplete after scaffolding and would ideally require accurate gap closure. Given that manual closure of incomplete regions with Sanger sequencing is expensive, genome assemblies submitted to sequence databases often lack a significant number of nucleotides, especially in the case of larger eukaryotes. Importantly, the missing repetitive or low-coverage regions may contain essential functions for the organism studied.

In this study we aim to reduce the number of undefined nucleotides by automatically filling in the gapped regions within scaffolds. For this purpose, paired reads are (re)used. In brief, our GapFiller method seeks to find read pairs of which one member matches within a sequence region and the second member falls (partially) within the gap. The latter reads are then used to close the gap through sequence (*k-mer*) overlap. Gaps are entirely closed only if the size of the sequence insertion corresponds closely to the estimated gap size after scaffolding, which is based on the alignment of paired reads to the contigs. The process is iteratively repeated until no further gaps can be closed. We demonstrate that GapFiller gives promising results both on bacterial and eukaryotic datasets and accurately closes most of the gaps. From a comparative analysis with IMAGE and SOAPdenovo we show that our algorithm provides the most reliable outcomes. Finally, we investigate the nature of the closed regions in the human genome and show that these contain important coding and non-coding information. The software is designed to be accessible to a broad audience given its user-friendly design and limited memory usage. GapFiller can be downloaded from [7].

## Results

### Findings on the bacterial datasets

GapFiller was evaluated on four distinct bacterial datasets that are characterized by different genome sizes, levels of complexity and short-read sequence input. Also, the assembly strategy underlying the construction of the scaffolds varies and provides some good practical examples of draft assemblies (see Materials and methods for more information). Quality assessment was performed through comparison with the known (closed) reference genome and error types were classified into three categories - SNPs, indels and misjunctions - according to [8]. A first observation on these datasets is that automated gap closure can indeed be an important step in finishing (bacterial) genomes. In Table 1 we show that all methods can effectively close a significant amount of gaps and also that the total gap length is drastically reduced. However, from the *Escherichia coli *and *Streptomyces coelicolor *datasets it is apparent that the IMAGE strategy clearly underperforms the other two strategies on most quality measurements: the total gap length is reduced at most by 60% even though the number of errors increases significantly. This is partly due to the fact that IMAGE can not handle multiple libraries (nor mate pair libraries), and therefore the analysis was performed using only the library with the shortest insert size. In contrast, SOAPdenovo and GapFiller show more promising results: in *E. coli *the number of gaps (gap count) can be reduced by 97% and 98%, respectively; in *S. coelicolor *these numbers are 62% and 85%, respectively. Overall, the results indicate GapFiller closes a larger amount of gaps while making significantly less errors than the other methods. Arguably, the total gap length is shorter after gap closure with SOAPdenovo, but apparently at the cost of an increased number of misassemblies. The latter two tendencies are more pronounced in the *Staphylococcus aureus *and *Rhodobacter sphaeroides *datasets. A likely explanation resides in the fact that the raw input FASTQ reads are heavily filtered with Quake [9], leading to a relatively low genome coverage. For comparison purposes we reran our GapFiller algorithm with less stringent settings (GapFiller-LC: min coverage *o *= 1, ratio *r *= 0.5) to yield a more similar gap length to SOAPdenovo. We observe that applying less conservative settings leads (obviously) to more errors, but that these are still significantly less than those made with SOAPdenovo (regardless of the error type).

**Table 1:** Gap closure results obtained on the bacterial datasets

Method	Original	IMAGE	SOAPdenovo	GapFiller	GapFiller-LC
*Escherichia coli*					
Genome size (bp)	4,478,287	4,530,961	4,490,973	4,490,638	
Scaffolds	179	179	179	179	
Gap count	544	291	16	11	
Total gap length (bp)	12,516	2,861	16	130	
Errors (SNPs)	12	40	33	22	
Errors (indels)	4	17	25	9	
Errors (misjoins)	1	1	1	1	
N50	50,557	50,558	50,558	50,558	
					
*Streptomyces coelicolor*					
Genome size (bp)	8,558,275	8,576,331	8,557,720	8,558,333	
Scaffolds	115	115	115	115	
Gap count	158	63	60	23	
Total gap length (bp)	9,221	4,009	1,288	806	
Errors (SNPs)	299	423	406	280	
Errors (indels)	664	677	769	686	
Errors (misjoins)	12	17	18	18	
N50	173,822	173,822	173,822	173,822	
					
*Staphylococcus aureus*					
Genome size (bp)	2,880,676		2,880,926	2,881,756	2,883,448
Scaffolds	19		19	19	19
Gap count	48		27	27	22
Total gap length (bp)	9,900		1,547	5,508	1,861
Errors (SNPs)	79		260	98	173
Errors (indels)	16		53	26	37
Errors (misjoins)	4		13	7	5
N50	1,091,731		1,091,333	1,092,281	1,092,421
					
*Rhodobacter sphaeroides*					
Genome size (bp)	4,609,785		4,609,466	4,609,596	4,610,796
Scaffolds	38		38	38	38
Gap count	170		163	161	139
Total gap length (bp)	21,409		14,166	20,667	17,625
Errors (SNPs)	218		410	230	300
Errors (indels)	187		294	190	199
Errors (misjoins)	6		10	6	7
N50	3,192,334		3,192,075	3,192,215	3,192,974

Gap closure results obtained on four bacterial datasets show that the GapFiller strategy yields the most accurate finished genomes. Also, the gap count is lower compared to the other methods. The IMAGE method significantly underperforms on all quality measures and would therefore not be the preferred method to use. Differences are smaller between GapFiller and SOAPdenovo. Interestingly, whereas the gap count after closure is generally less for GapFiller, SOAPdenovo yields in three cases a shorter total gap length. This suggests the latter method is able to close larger gaps. Strikingly, however, the amount of errors is significantly higher for SOAPdenovo regardless of the source (SNPs, indels and misjoins). Even when applying less strict settings for GapFiller (GapFiller-LC: minimum coverage *o *= 1, ratio *r *= 0.5) to shorten the total gap length, our method still yields significantly less errors.

**Figure 1:**
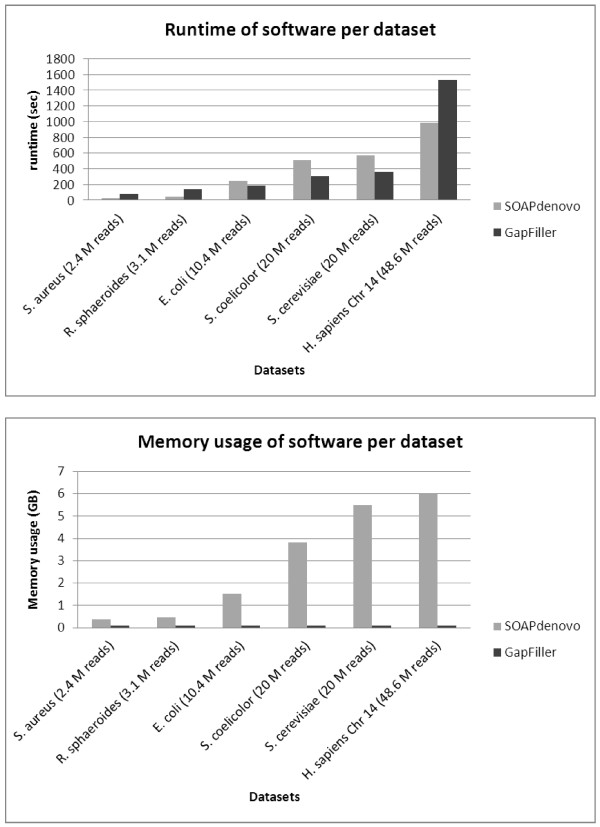
**Time and memory consumption of gap closure software**. Comparative analysis of the runtime and memory usage per dataset based on a single iteration. SOAPdenovo needs a shorter time to complete the analysis if the amount of data is very small or large, whereas GapFiller is faster for intermediate data sizes (10 to 20 million reads). With regard to memory usage, GapFiller outperforms SOAPdenovo since intermediate output is temporarily stored (and not kept in the memory). For all datasets analyzed, GapFiller requires only 0.1 GB of memory, which is mostly consumed by the Burrows-Wheeler Aligner (BWA). Note that no results are displayed for IMAGE since the method can not handle multiple libraries and requires very large computation times to complete the process. M, million.

Another interesting observation concerns the computational resources needed to run the software. Figure 1 shows a comparative analysis between the runtimes and memory usage of SOAPdenovo and GapFiller based on one iterative cycle. Apparently SOAPdenovo can complete the process faster if the number of input reads is very low or high, but for intermediate data sizes (10 to 20 million reads) GapFiller needs somewhat less time. Differences are more pronounced when comparing the memory usage of both methods: SOAPdenovo requires an increasing amount of memory to analyze larger input datasets (up to 3.8 GB for *S. coelicolor *and 6.0 GB for human) whereas GapFiller shows stable memory usage of approximately 0.1 GB. This is because GapFiller seeks to store the intermediate output temporarily rather than maintaining it in the memory. As a consequence the method is well suited for computing systems with smaller amounts of memory.

Summarizing, we provide strong evidence that bacterial draft assemblies can be accurately closed using an automated strategy. The few gaps that are left open by GapFiller mainly concern more difficult repeated areas. Here the extension method finds multiple alternatives with an equal ratio. These regions can eventually be closed through additional Sanger sequencing using only a limited number of reactions.

### Gap closure in a small and large eukaryote: analysis of the yeast model organism *Saccharomyces cerevisiae *and the human chromosome 14

*Saccharomyces cerevisiae *is perhaps the most commonly used yeast and plays an important role in fermentation of beer and bread. We used the 288c reference genome to simulate two paired-end libraries, which were subsequently used for assembly (see Materials and methods for more specifications). In Table 2 we show that GapFiller can also be of value in closing genomes of smaller eukaryotes such as yeast: the number of gaps can be reduced with 84% (against 76% for SOAPdenovo) at a relatively low error rate. Nonetheless, as observed also for the bacterial datasets, SOAPdenovo more effectively reduces the total gap length (95% against 85% for GapFiller). The IMAGE method was not evaluated on eukaryotic datasets due to the very large computation time needed.

**Table 2:** Gap closure results obtained on the eukaryotic datasets

	Method		
	
	Original	SOAPdenovo	GapFiller
*Saccharomyces cerevisiae*			
Genome size (bp)	11,388,647	11,388,600	11,388,609
Scaffolds	334	334	334
Gap count	283	67	45
Total gap length (bp)	19,358	994	2,873
Errors (SNPs)	890	1,033	931
Errors (indels)	565	754	648
Errors (misjoins)	23	42	31
N50	84,640	84,640	84,649
			
*Homo sapiens *(chromosome 14)			
Genome size (bp)	95,081,274	95,059,687	95,072,801
Scaffolds	19,249	19,249	19,249
Gap count	2,820	1,986	1,682
Total gap length (bp)	949,137	423,107	699,550
Errors (SNPs)	76,653	79,266	76,928
Errors (indels)	21,261	23,144	22,338
Errors (misjoins)	179	224	187
N50	7,748	8,262	8,469

Results of SOAPdenovo and GapFiller obtained for the *S. cerevisiae *and human genome show the suitability of both methods to close gaps also in eukaryotic genomes. Patterns are similar to the observations made for bacteria: overall, GapFiller yields the most reliable results and the lowest gap count whereas SOAPdenovo yields a significantly shorter total gap length (though at the cost of a fairly increased error rate). In human the shortened genome size and total gap length obtained by SOAPdenovo (together with the increased indel and misjoin error rate) might indicate that some gaps are eventually closed by collapsing of (repeated) elements.

Similar tendencies are observed for human chromosome 14 (see Materials and methods for information about the datasets used), even though the amount of data and chromosome size differ largely in comparison to the yeast dataset. As expected, the number of scaffolds is higher for human, but notably the average scaffold length is relatively short (which is likely due to the use of ABySS for creation of the draft assembly). Consequently, the outcomes are less impressive, but still there seems to be an inverse relationship between the gap count (-29% after running SOAPdenovo, -40% with GapFiller) and the total gap length (-55% with SOAPdenovo, -26% with GapFiller). Given the observation that, after gap closure with SOAPdenovo, the total genome size is significantly reduced whereas the number of errors due to indels and misjoins has increased, it might be argued that a portion of these closures can be attributed to collapsing of (repeated) elements.

From a practical point of view it should be observed that both methods can run well on small scale systems: SOAPdenovo appears to be faster on larger input sets whereas GapFiller uses significantly less memory (Figure 1).

### Functional analysis of the human chromosome 14

To further analyze the nature of the closed region, we extracted all annotations from the human GRCh37 chromosome 14 [GenBank:NC000014] and then aligned the corresponding sequences to the scaffolds (before and after closure with GapFiller). For each annotation type we counted the number of gapped nucleotides that were closed with GapFiller. Results are provided in Table 3 and show that novel nucleotides are incorporated in a wide range of functional regions, showing that gap closure adds mostly functional residues to the assembly. The largest relative increase of nucleotides is seen in coding regions (38.67% for genes, 30.53% for mRNA and 26.89% for coding sequences). Overall, the method is applicable on the relatively large genomic spectrum and does not just solve regions that are bound to specific functional characteristics.

**Table 3:** Functional properties of gap closed regions

Annotation type	Total size in NC_000014 (bp)	Nucleotides closed with GapFiller (bp)	Portion of total nucleotides closed (%)
Gene	38,883,890	33,969	38.67%
mRNA	34,180,962	26,818	30.53%
CDS	29,380,348	23,617	26.89%
Other RNA	3,245,865	2,755	3.14%
V-segment	78,961	655	0.75%
C-region	36,984	23	0.03%
ncRNA	11,447	0	0%
			
Total	105,818,457	87,837	100.00%

Functionalities associated with the nucleotides incorporated with GapFiller based on the annotation of human chromosome 14. Clearly the method can add valuable sequence information to a number of functionalities present in GenBank:NC_000014. Most nucleotides are incorporated in coding sequence (CDS) and mRNA regions. ncRNA, non-coding RNA.

## Discussion

Dramatic advances in sequencing technologies have opened new possibilities for whole genome analysis. The increasing read length of next-generation sequencing platforms, as well as the promising perspectives of third generation sequencing platforms, will inevitably lead to better assemblies and represent genomes in large stretches of DNA. Also, third generation technologies (such as the PacBio and IonTorrent systems) will be capable of outputting sequencing reads with large undefined inserts, thus providing valuable paired read information for the assembly and scaffolding process. Concurrently, the development of genome closure software should also receive attention to overcome difficult genomic regions that cannot be covered by draft assemblies.

Our results with GapFiller indicate that gapped genomics regions can be reliably closed through an automatic protocol that uses only short sequencing reads. Costly Sanger sequencing can therefore be limited to a few difficult repeat areas. Also, we show that the method is suited for both bacterial and (large) eukaryotic datasets in terms of accuracy, time and memory usage. In the human dataset we underscore that gapped regions may contain diverse but crucial functional information, which is missed in the draft assembly.

From in-depth investigation of closed regions with GapFiller and SOAPdenovo, we argue that our GapFiller method has three main advantages. First, it takes into account the estimated gap size prior to closure, thus discarding erroneous closures that are shorter or larger than expected. Second, it does not try to fill a gap through local assembly of reads into contigs, but rather seeks to extend the contigs from each end through *k*-mer overlap. The latter point is especially important to overcome short tandem repeats. And third, it takes into account the contig edges, often a source of misassemblies, and re-evaluates these during gap closure. Importantly, GapFiller requires only limited computational resources and thus is also suited for (larger) eukaryotic genomes. Also, SOAPdenovo shows a good performance in terms of speed and memory usage and appears a valuable alternative to our strategy. From the results obtained on both bacterial and eukaryotic datasets, it appears SOAPdenovo is better able to close larger gaps. The explanation might reside in the fact that GapFiller implements a more conservative strategy than SOAPdenovo. Given that larger gaps usually represent more complex regions than smaller gaps, GapFiller may be simply more careful in avoiding dubious closures. This is also in line with the observation that GapFiller yields generally more accurate results than SOAPdenovo. Alternatively, a different explanation for GapFiller being less effective in closing larger gaps could be provided by the first advantage mentioned above. If the estimated gapsize in the scaffold does not meet the size of the closed fragment, such closure will be rejected. However, from a practical point of view it is still relatively difficult to estimate the exact gap size based on next-generation sequencing paired-read libraries. In particular, mate pair libraries can show a relatively large insert distribution, thus making it hard to define the correct distance between the pairs. Also paired-end background noise in mate pair data is commonly observed and leads to incorrect scaffolds. Consequently, there is a possibility that a correct closure is rejected because of the erroneous gap size estimation after scaffolding. Future research will hopefully lead to better insight into these issues.

The IMAGE method instead has several limitations in terms of quality, speed and ease of use (the method can handle only one library per analysis). Notably, the strategy produces relatively large genomes, and this is most likely a consequence of sequence extension of the left and right scaffold edges.

The findings in this paper have been derived using different short-read Illumina libraries. The length of the gapped regions is limited to the insert distance of paired reads. Nonetheless, the promising outcomes strongly indicate that long-range gaps can also be effectively closed with high quality 454 and/or single molecule paired sequence reads. Also, we have put emphasis on creating a user-friendly and fast tool so that it can be of wide use for the community. We feel GapFiller can make a significant contribution to (almost) closing genome assemblies in a reliable manner.

## Materials and methods

### GapFiller algorithm

**Figure 2:**
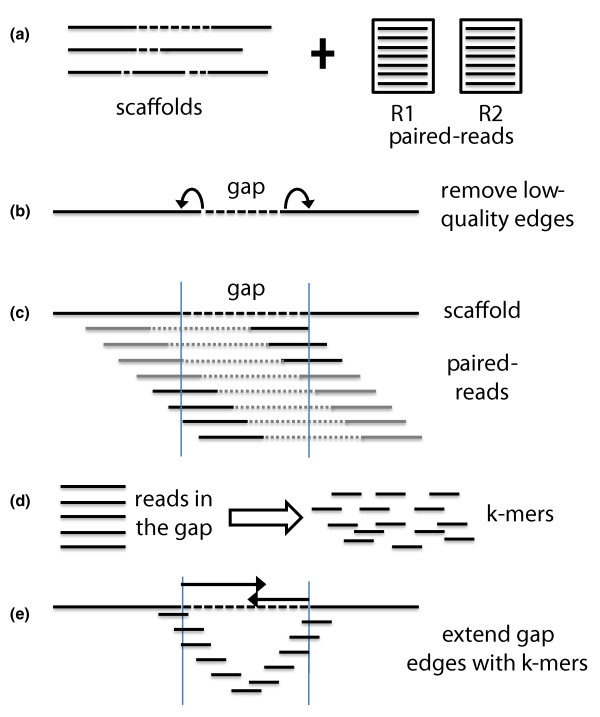
**Schematic overview of the GapFiller algorithm**. **(a) **The input data consist of a set of scaffold sequences containing gapped nucleotides and one or more sets of paired-end and/or mate-pair reads. **(b) **As a pre-processing step low quality nucleotides are removed from the sequence edges, thus enlarging the gap of ten nucleotides from each side. It should be stressed that the contig ends resulting from a draft assembly often contain misassemblies. **(c) **Paired-reads are aligned to the scaffolds and retained if one pair aligns to a scaffold sequence (dark grey) and one pair to a gapped region (black). **(d) **All pairs that are estimated to fall in the gapped regions are split into *k*-mers and used for gap filling. **(e) **The gap is closed from each edge by using *k*-mers that present a sequence overlap of size (*k*-mer - 1) and one nucleotide overhang. Gaps are closed if the right and left extensions can be merged and correspond to the estimated sequence gap.

The GapFiller method works as follows (see Figure 2 for a schematic overview). The input consists of a set of scaffold sequences (in FASTA format) with gapped nucleotides represented as Ns and one or multiple sets of paired reads (in FASTQ or FASTA format). The data handling and analysis are setup in a multithreaded manner, thus allowing multiple datasets (libraries) to be processed simultaneously (by default one thread is used, but for this study we set this to eight). In a pre-filtering step the nucleotides at the edges of each gap are trimmed off (parameter *t*, default = 10 nucleotides) since these often contain misassemblies. Then all pairs are aligned to the trimmed input scaffolds with Bowtie [10] or the Burrows-Wheeler Aligner (BWA) [11]. The alignment method can be selected by the user, but we recommend using BWA in case of long reads. Bowtie instead is more suited for shorter reads, also because of the decreased runtime. For this study we used BWA on all datasets. Pairs are only kept if a) one read can be aligned and b) the second read (partially) falls within a gapped region. The latter is calculated based on the expected distance between the read pairs: the maximum allowed deviation used for this study was 25% of the pre-defined distance between the pairs. The final set of reads, which is expected to be (partially) in a gapped region, is then split into shorter *k*-mers that are used to gradually close the gap from each edge. The *k*-mer size is set by default to 30 nucleotides (1 *+ m*, where m corresponds to a default sequence overlap of 29 nucleotides). Gaps are iteratively filled from the left and right edge by incorporating one overhang nucleotide at a time, provided the position is sufficiently covered (parameter *o*, default = 2). In case of ambiguous overhang nucleotides (that is, in a situation of allelic differences), a majority voting ratio is applied (parameter *r*, default = 0.7). If a nucleotide is incorporated, the *k*-mer(s) used for extension are removed and the algorithm checks if the gap can be closed. Gaps are only considered to be closed if a) an overlap can be found between the two extensions (parameter *n*, default = at least 10 nucleotides) and b) the sequence insertion corresponds to a pre-estimated gap length in the scaffold (parameter *d*, default = maximally 50 nucleotides difference). Otherwise, the next iteration is started. If no overhang nucleotides can be incorporated anymore, the whole process is repeated a second time by re-using all *k*-mers. Finally, the user can decide to repeat the complete analysis (alignment and gap closure) on the novel assembly. The number of such 'global' iterations can be specified using the (parameter *i*, default = 10).

### Short-read datasets

To evaluate the results of our algorithm, we used short-read Illumina data from *E. coli, S. coelicolor, S. aureus, R. sphaeroides, S. cerevisiae *and *Homo sapiens*. Our aim was to create a divergent benchmark using a variety of organisms, dataset types and assembly strategies. For *E. coli *the dataset consists of a paired-end library taken from the NCBI Short Read Archive [SRA:SRR001665] (insert size of 200 bp, 36 cycles, 10.4 million reads). The dataset of *S. coelicolor *and *S. cerevisiae *consist of simulated paired-end libraries constructed from reference genomes deposited in GenBank. Random paired-end reads were created using wgsim (at default settings), which is part of the SAM tools package [12]. For *S. coelicolor *we used the A3(2) reference genome [GenBank:AL645882] to construct two paired-end libraries (insert sizes of 200 and 500 bp, 70 cycles, 10 million reads per library, whereas for *S. cerevisiae *we used all chromosomes of the S288c genome [GenBank:NC001133 to NC_001148] to construct two paired-end libraries (insert sizes of 200 and 500 bp, 70 cycles, 20 million reads per library). For *S. aureus, R. sphaeroides *and *H. sapiens *(chromosome 14) quakeCor-filtered [9] short-read datasets were taken from the Genome assembly Gold-standard Evaluations website [13]. For *S. aureus *this included a paired-end (insert size of 180 bp, 88 cycles, 0.8 million reads) and a mate-pair library (insert size of 3,500 bp, 37 cycles, 1.6 million reads). Similarly, for *R. sphaeroides *this also included a paired-end (insert size of 180 bp, 76 cycles, 1.5 millino reads) and a mate-pair library (insert size of 3,500 bp, 72 cycles, 1.5 million reads). For *H. sapiens *(chromosome 14) the dataset included one paired-end library (insert of 155 bp, 93 cycles, 32.6 million reads) and two mate-pair libraries (shortjump library: insert size of 2,283 to 2,803 bp, 82 cycles, 14.5 million reads; longjump library: insert size of 35,295 to 35,318 bp, 79 cycles, 2.0 million reads).

### Assembly and scaffold construction

For three organisms a draft assembly was made using the paired-end libraries. For *E. coli *we used SOAPdenovo v1.3 [3] whereas for *S. coelicolor *and *S. cerevisiae *we used the CLCbio *de novo *assembler v4.9. Scaffolds were produced using the same libraries as follows. For *E. coli *the 1,111 contigs of the draft assembly were linked into 179 scaffolds using SSPACE Premium v1.0 [4]. For the *S. coelicolor *and *S. cerevisiae *genomes a similar strategy was adopted resulting in 115 scaffolds (430 contigs) and 334 scaffolds (733 contigs), respectively. For the remaining three species, *S. aureus, R. sphaeroides *and *Homo sapiens*, assemblies were retrieved from the GAGE website [13]. Based on the outcomes of the comparative analysis, we decided for the bacterial species to use the scaffolds produced with ALLPATHS-LG strategy [14] and for human the scaffolds (≥ 200 bp) produced with ABySS [2].

### IMAGE and SOAPdenovo gap closure software

Comparative analysis with IMAGE [6] was performed using version 2.3, which was downloaded from [15]. The program was run at default settings using a *k*-mer size of 31 and with 10 iterations. The insert size of the paired-end libraries was specified accordingly. SOAPdenovo gap closer software version 1.10 was downloaded from [16]. The program was run at default settings using a *k*-mer size of 30 and in total 8 threads.

### Quality assessment of assemblies

In order to assess the quality of the assemblies, we followed the criteria set by [8]. Errors were classified into three categories: SNPs, indels (including indels ≤ 5 bp and indels > 5 bp) and misjoins (including inversions, relocations and translocations).

### Compute server specifications

All analyses were performed on a 32 GB Linux machine (Intel Xeon X7350, 2.93 GHz).

### Availability of software

The software can be downloaded from [7]and is free of charge for academic users.

## Abbreviations

bp: base pair; BWA: Burrows-Wheeler Aligner; LC: low coverage; SNP: single nucleotide polymorphism.

## Competing interests

The authors declare that they have no competing interests.

## Authors' contributions

MB conceived of the study and wrote the program, WP participated in its design and coordination and wrote the manuscript. All authors read and approved the final manuscript.
